# Correlation between obstructive sleep apnea and weight-adjusted-waist index: a cross-sectional study

**DOI:** 10.3389/fmed.2024.1463184

**Published:** 2024-10-23

**Authors:** Mengye Zhang, Xiaolu Weng, Jing Xu, Xue Xu

**Affiliations:** ^1^Department of Rehabilitation Medicine, The First Affiliated Hospital of Zhejiang Chinese Medical University (Zhejiang Provincial Hospital of Chinese Medicine), Hangzhou, China; ^2^Department of Endocrinology, The Second Affiliated Hospital and Yuying Children's Hospital of Wenzhou Medical University, Wenzhou, China

**Keywords:** obesity, WWI, waist circumference, OSA, weight

## Abstract

**Background:**

Obesity is recognized as a prominent factor in the pathogenesis of obstructive sleep apnea (OSA). The weight-adjusted-waist index (WWI) has emerged as a novel metric for assessing adiposity. The study aimed to investigate the potential correlation between WWI and OSA.

**Methods:**

In this study, a cross-sectional analysis was conducted on the data from the National Health and Nutrition Examination Survey (NHANES) during the period from 2013 to 2020. To examine the correlation between WWI and OSA, multivariate logistic regression, smooth curve fitting, subgroup analysis and receiver operating characteristic (ROC) curve were employed.

**Results:**

Among the total 18,080 participants, 9,050 were categorized as having OSA. It was observed that as the quartile range of WWI increased, there was a gradual rise in the prevalence of OSA (37.4% vs. 50.3% vs. 55.1% vs. 57.5%, *p* < 0.001). Logistic regression analyses demonstrated a significant positive correlation between WWI and the risk of OSA, even after accounting for potential confounders (OR = 1.314, 95% = 1.223, 1.411). Subgroup analysis further revealed a stronger correlation between OSA and WWI among individuals with normal weight, those under 50 years old, and those engaging in moderate physical activities. Smooth curve fitting identified a positive non-linear correlation, with an inflection point at 11.678. ROC analysis indicated that WWI (AUC = 0.664) can serve as a more robust predictor for OSA compared to BMI and waist circumference.

**Conclusion:**

This study provides evidence that elevated levels of WWI are correlated with an increased risk of OSA, indicating the potential utility as predictive indicators for OSA.

## Introduction

OSA is characterized by the complete or partial collapse of the upper airway for a minimum of 10 s during sleeping, leading to complete cessation (apnea) or decreased airflow (hypoventilation). Excessive daytime drowsiness is a key symptom of OSA (1, 2). Epidemiological data suggest that OSA affects around 34% of males and 17% of females aged 30 to 70 years old in the United States (3). If the rest is untreated, OSA may result in serious health complications such as cardiovascular diseases (4–6), hypertension (7) and diabetes mellitus (8). Consequently, the identification of novel and precise biomarkers for the early detection of OSA is imperative.

Obesity stands out as a significant risk factor for OSA (9, 10), with its weight fluctuations affecting the severity of conditions (11). In clinical practice, various standard anthropometric measures are utilized to assess obesity and its related risks, each possessing inherent constraints. BMI and waist circumference (WC) are commonly used tools for assessing obesity, but they have limitations in distinguishing fat type or distribution and muscle mass (12). Additionally, WC is not a reliable predictor of visceral adipose tissue at the individual level (12). “Obesity paradox” has been observed in various populations, with inconsistent or contradictory results between these traditional indicators of obesity (13). Therefore, WWI, a novel obesity metric, proposed by Park et al. in 2018, has emerged as a promising alternative (14). WWI, calculated as WC divided by the square root of body weight, retains the advantages of WC while reducing its correlation with BMI (15). WWI provides a nuanced distinction between muscle mass and fat, focusing primarily on abdominal obesity regardless of overall body weight (15, 16). Recent research suggests that an increase in WWI is a significant risk factor for metabolic disorders such as non-alcoholic fatty liver and diabetes mellitus (16–19).

However, the correlation between WWI and OSA remains unclear. This study aims to explore the correlation between WWI and the prevalence of OSA according to the data from NHANES. Additionally, it compared the correlation between BMI, WC and the prevalence of OSA, thereby evaluating the strength of the correlation between WWI and OSA.

## Methods

### Research population and research design

NHANES survey, conducted by NCHS (20), is a comprehensive study designed to assess the correlation between health promotion, nutrition, and disease prevention. The survey is conducted in every 2 years by taking interviews, physical examinations, and various sections covering dietary, demographic, examination, questionnaire data, and laboratory. Additional information regarding the NHANES database can be found at http://www.cdc.gov/nhanes.

**Figure 1:**
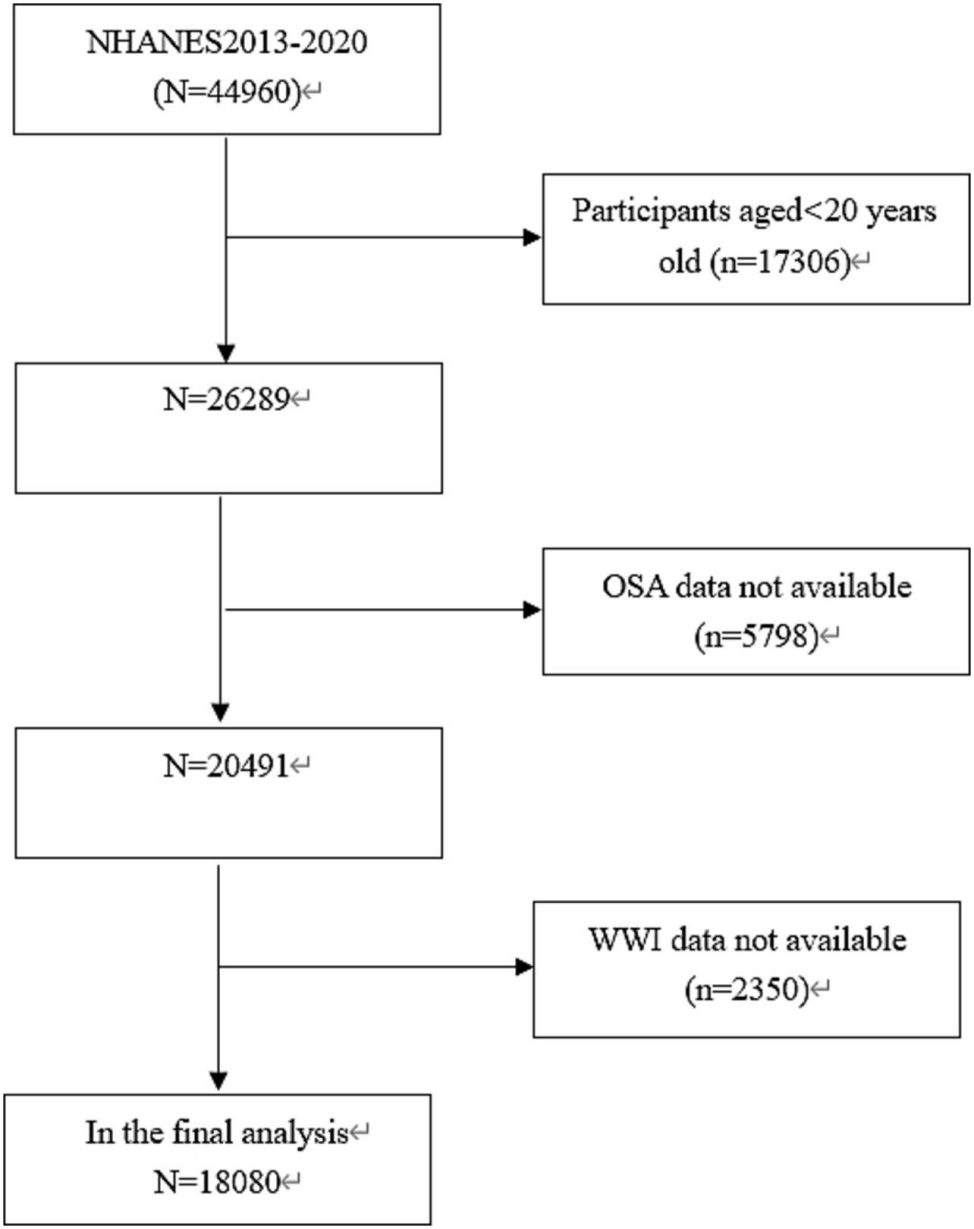
Flowchart of the sample selection from the 2013–2020 NHANES.

A total of 44,960 participants were included in the NHANES database from 2013 to 2020. Through rigorous exclusion and inclusion criteria, this study identified a sample size of 18,080 American adults from the NHANES during the period from 2013 to 2020. To ensure the relevance and accuracy of the study, specific exclusion criteria were applied, and 17,306 participants younger than 20 years old were excluded. Additionally, 5,798 individuals missing OSA data, and 2,350 participants missing WWI data were excluded (Figure 1).

### Assessment of OSA

Consistent with the prior research, OSA was diagnosed when an individual affirmed a positive response to at least one of the three questions in NHANES outlined as follows (21): (1) Daylight sleepy characterized by excessive drowsiness during waking hours despite receiving a minimum of sleep for 7 h per night, reporting between 16 to 30 times; (2) Snoring or cessation of breathing: Episodes of snorting, gasping or cessation of breathing occurring three or more times per week; (3) Snoring: Snoring on three or more occasions per week.

### Measurement of covariates

With previous studies as the basis (22, 23), potential confounding factors related to WWI and OSA were included in the final analysis, including demographic variables (age, height, race, blood pressure, gender, WC, educational attainment, weight, and physical activity), as well as questionnaire data on alcohol consumption, smoking status, hypertension, and diabetes mellitus. TC, FPG, LDL-C, ALT, UA, TG, GGT, AST, creatinine, albumin and HDL-C were collected in blood samples. Detailed measurement methodology and data acquisition for each variable can be accessed at www.cdc.gov/nchs/nhanes. In this study, WWI was calculated with the formula WC (cm) divided by the square root of weight (kg) (14).

### Statistical analysis

WWI was categorized into quartiles (Q1: ≤10.56; Q2: 10.56–11.14; Q3: 11.14–11.73; Q4: ≥11.73). Categorical characteristics were expressed as proportions, and continuous variables were summarized with means and standard errors. Discrepancies among quartile groups were assessed with Kruskal-Wallis H test for continuous variables and chi-square tests for categorical variables. The odds ratios (ORs) and 95% confidence intervals (CIs) between OSA and WWI were investigated with multiple logistic regression models. Significant variables identified in the univariate analysis were included in the multivariate analysis. Three models were utilized in the analysis: Model 1 (unadjusted), Model 2 (adjusted for gender and age only), and Model 3 (fully adjusted for drinking, BMI, smoking, gender, educational level, age, race, moderate physical activities, TG, diabetes mellitus, albumin, hypertension, FPG, TC, ALT, HDL-C, AST, creatinine, GGT and uric acid). Potential modifications were investigated to this correlation by covariates through interaction and subgroup analyses. Furthermore, the non-linear correlation between OSA and WWI was assessed with smooth curve fitting utilizing generalized additive models. For non-linear models, the two-piecewise linear regression models were performed on both parts of the inflection point with a recursive algorithm for the non-linearity was detected. AUC was calculated through ROC analysis to evaluate the diagnostic ability of WWI, BMI and WC for identification of OSA. A significance threshold (*p* < 0.05) was applied for all statistical analysis. Data were analyzed with the statistical software programs R (version 3.4.3) and EmpowerStats (version 2.0) to examine the correlation between WWI and OSA.

## Results

### Baseline characteristics of subjects

**Figure 2:**
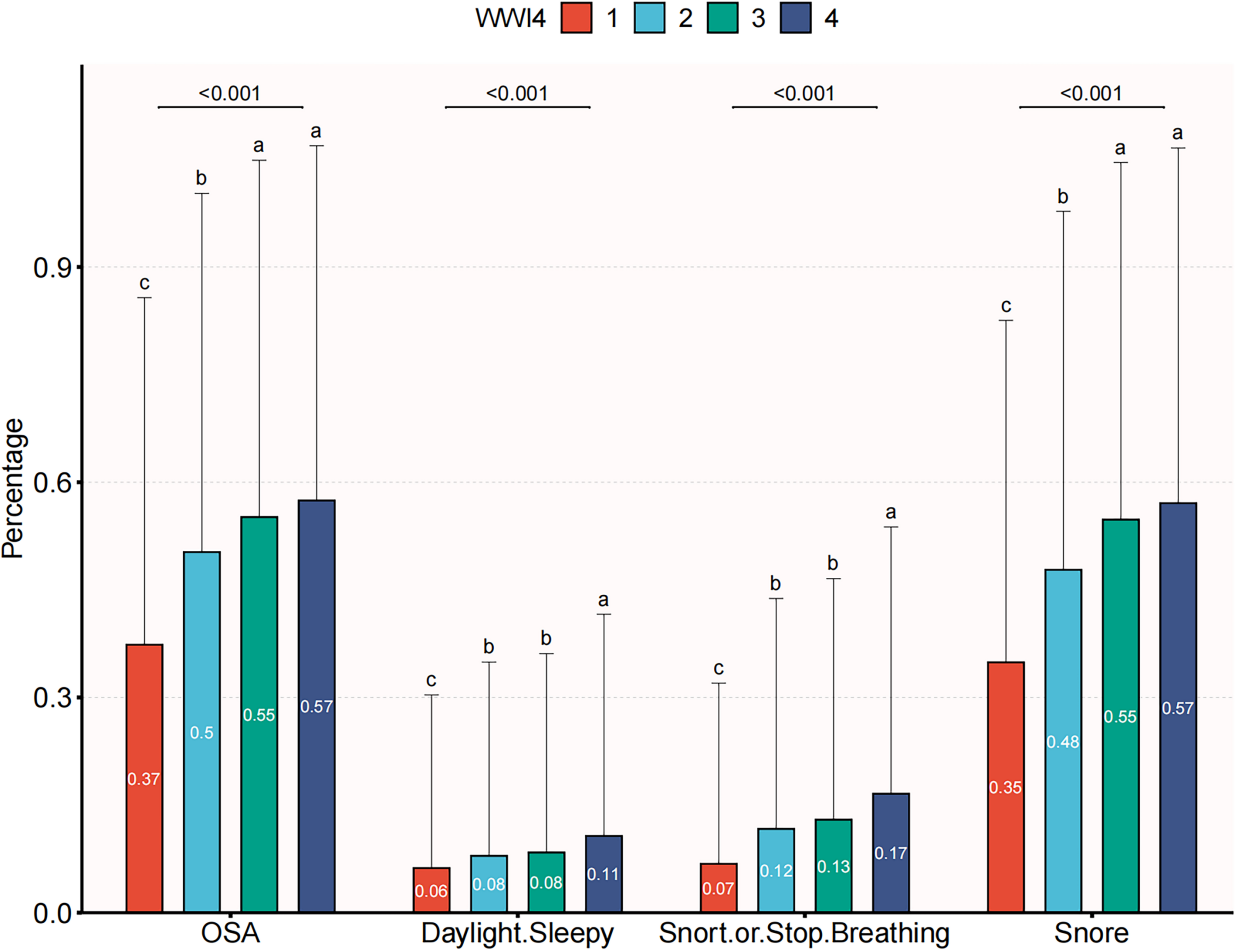
The prevalence of OSA and OSA symptom across quartiles of WWI.

A total of 18,080 subjects ranging from 20 to 80 years old were included in this study, with a prevalence of OSA of 50.1%. Demographic characteristics, stratified by WWI quartiles, are presented in Table 1. Subjects in the highest WWI quartile exhibited a higher prevalence of OSA, diabetes mellitus, hypertension and elevated levels of age, AST, weight, uric acid, BMI, TG, SBP, waist circumference, ALT, DBP, FPG and TC compared to those in the lowest quartile. Conversely, individuals in the highest quartile had lower levels of HDL-C and albumin (*p* < 0.01) (Table 1). As illustrated in Figure 2, the prevalence of OSA increased across quartiles: 37.4% in Q1, 50.3% in Q2, 55.1% in Q3, and 57.5% in Q4, along with a rise in OSA symptoms such as daytime sleepiness, snoring, and apnea.

**Table 1:** Weighted characteristics of the study population based on WWI quartiles.

Characteristic	Q1	Q2	Q3	Q4	*p* value
Number	4,519	4,520	4,521	4,520	
Age, year	38.8 ± 14.6	48.1 ± 15.7	54.5 ± 16.3	59.8 ± 15.8	<0.001
Race, *n*%					<0.001
Mexican American	367 (8.1)	574 (12.7)	786 (17.4)	751 (16.6)	
Other Hispanic	1,466 (32.4)	1,066 (23.6)	981 (21.7)	854 (18.9)	
Non-Hispanic White	1,381 (30.6)	1,468 (32.5)	1,510 (33.4)	1821 (40.3)	
Non-Hispanic Black	389 (8.6)	510 (11.3)	510 (11.3)	556 (12.3)	
Other race	916 (20.3)	902 (20)	734 (16.2)	538 (11.9)	
Moderate activities, *n*%					<0.001
Yes	2,360 (52.2)	1948 (43.1)	1749 (38.7)	1,393 (30.8)	
No	2,158 (47.8)	2,571 (56.9)	2,770 (61.3)	3,125 (69.2)	
Diabetes, *n*%					<0.001
Yes	139 (3.1)	487 (10.8)	784 (17.3)	1,279 (28.3)	
No	4,380 (96.9)	4,033 (89.2)	3,737 (82.7)	3,241 (71.7)	
Hypertension					
Yes	795 (17.6)	1,472 (32.6)	1971 (43.6)	2,511 (55.6)	
No	3,724 (82.4)	3,048 (67.4)	2,550 (56.4)	2009 (44.4)	
Education level, *n*%					<0.001
Less than high school	562 (12.4)	804 (17.8)	1,028 (22.7)	1,213 (26.8)	
High school or above	3,957 (87.6)	3,716 (82.2)	3,493 (77.3)	3,307 (73.2)	
Drinking, *n*%					<0.001
Current or ever	2,900 (64.2)	2,809 (62.1)	2,790 (61.7)	2,670 (59.1)	
Never	1,619 (35.8)	1711 (37.9)	1731 (38.3)	1850 (40.9)	
Smoking, *n*%					<0.001
Current or ever	1,666 (36.9)	1907 (42.2)	2025 (44.8)	1975 (43.7)	
Never	2,849 (63.1)	2,612 (57.8)	2,493 (55.2)	2,542 (56.3)	
Male, *n*%	2,691 (59.5)	2,463 (54.5)	2,191 (48.5)	1,452 (32.1)	<0.001
OSA, *n*%	1,688 (37.4)	2,272 (50.3)	2,493 (55.1)	2,597 (57.5)	<0.001
Weight, cm	74.5 ± 17.8	82.0 ± 20.7	85.2 ± 22.2	89.4 ± 25.0	<0.001
Body mass index, Kg/m^2^	25.3 ± 5.2	28.8 ± 5.9	30.8 ± 6.6	34.1 ± 7.8	<0.001
Waist circumference, cm	86.0 ± 11.2	97.6 ± 12.3	104.5 ± 13.5	114.6 ± 16.1	<0.001
Systolic blood pressure, mmHg	118.9 ± 15.8	124.4 ± 18.3	128.6 ± 19.3	131.5 ± 19.4	<0.001
Diastolic blood pressure, mmHg	70.3 ± 11.7	72.8 ± 12.1	71.7 ± 13.6	69.1 ± 14.7	<0.001
FPG, mmol/L	5.6 ± 1.3	6.2 ± 1.9	6.5 ± 2.3	6.9 ± 2.6	<0.001
ALT, U/L	21.5 ± 19.1	24.4 ± 17.9	24.2 ± 17.6	22.6 ± 16.1	<0.001
AST, U/L	23.1 ± 15.9	23.2 ± 12.1	23.2 ± 13.8	22.8 ± 17.1	0.431
GGT, U/L	24.9 ± 39.2	33.1 ± 48.5	33.3 ± 46.1	33.2 ± 53.3	<0.001
Albumin, g/dL	4.26 ± 0.34	4.17 ± 0.34	4.10 ± 0.34	3.99 ± 0.35	<0.001
Creatinine, umol/L	78.0 (65.0, 90.0)	74.0 (63.0, 88.0)	73.0 (60.0, 88.0)	71.0 (59.0, 88.0)	<0.001
Uric acid, umol/L	303.3 (249.8, 356.9)	315.2 (255.8, 380.7)	321.2 (261.7, 380.7)	321.2 (267.7, 386.6)	<0.001
Total cholesterol, mmol/L	4.74 ± 1.01	4.98 ± 1.06	4.97 ± 1.12	4.85 ± 1.10	<0.001
Triglycerides, mmol/L	0.98 (0.70, 1.46)	1.31 (0.91, 2.00)	1.42 (1.02, 2.08)	1.56 (1.12, 2.21)	<0.001
HDL-cholesterol, mmol/L	1.50 ± 0.44	1.38 ± 0.43	1.35 ± 0.43	1.32 ± 0.38	<0.001
LDL-cholesterol, mmol/L	2.75 ± 0.87	2.93 ± 0.90	2.93 ± 0.99	2.80 ± 0.93	<0.001
WWI	10.03 ± 0.42	10.86 ± 0.16	11.42 ± 0.17	12.23 ± 0.41	<0.001

Values are mean ± SD or number (%). *p* < 0.05 was deemed significant. BMI, body mass index; FPG, fasting blood glucose; TC, total cholesterol; TG, triglyceride; HDL-c, High density lipoprotein cholesterol; LDL-c, Low density lipoprotein cholesterol; GGT, glutamyl transpeptidase; WWI, Weight-Adjusted-Waist Index.

### Correlation between WWI and OSA

In order to explore the correlation between OSA and WWI, three multiple regression models were developed (Table 2). Model 1, the unadjusted model, indicated a statistically significant positive correlation between OSA and WWI, which remained evident after adjusting for all covariates in Model 3 (OR = 1.314, 95% CI: 1.223, 1.411, *p* < 0.001). Furthermore, WWI was categorized into quartiles for sensitivity analysis. In the fully adjusted Model 3, participants in the second, third, and fourth quartiles demonstrated a statistically significant increase in the risk of OSA by 39.5, 71.4, and 73.8%, respectively, compared to those in the lowest quartile, with statistical significance.

**Table 2:** Association between WWI and OSA in logistic regression analysis.

	Model1 OR (95% CI), *p* value	Model II OR (95% CI), *p* value	Model III OR (95% CI), *p* value
WWI	1.429 (1.380, 1.480), <0.001	1.550 (1.487, 1.616), <0.001	1.314 (1.223, 1.411), <0.001
WWI(Quartile)			
Q1	Reference	Reference	Reference
Q2	1.695 (1.559, 1.843), *p* < 0.001	1.744 (1.600 ~ 1.902), *p* < 0.001	1.395 (1.218, 1.598), *p* < 0.001
Q3	2.062 (1.895, 2.243), *p* < 0.001	2.187 (1.997 ~ 2.394), *p*< 0.001	1.714 (1.481, 1.984), *p* < 0.001
Q4	2.265 (2.082, 2.464), *p* < 0.001	2.600 (2.360 ~ 2.865), *p* < 0.001	1.738 (1.476, 2.047), *p* < 0.001
P for trend	<0.001	<0.001	<0.001

Model I: None covariates were adjusted; Model II: gender and age were adjusted; Model III: drinking, BMI, smoking, gender, educational level, age, race, moderate physical activity, TG, diabetes, albumin, hypertension, FPG, TC, ALT, HDL-C, AST, creatinine, GGT and uric acid were adjusted.

### Subgroup analysis

**Figure 3:**
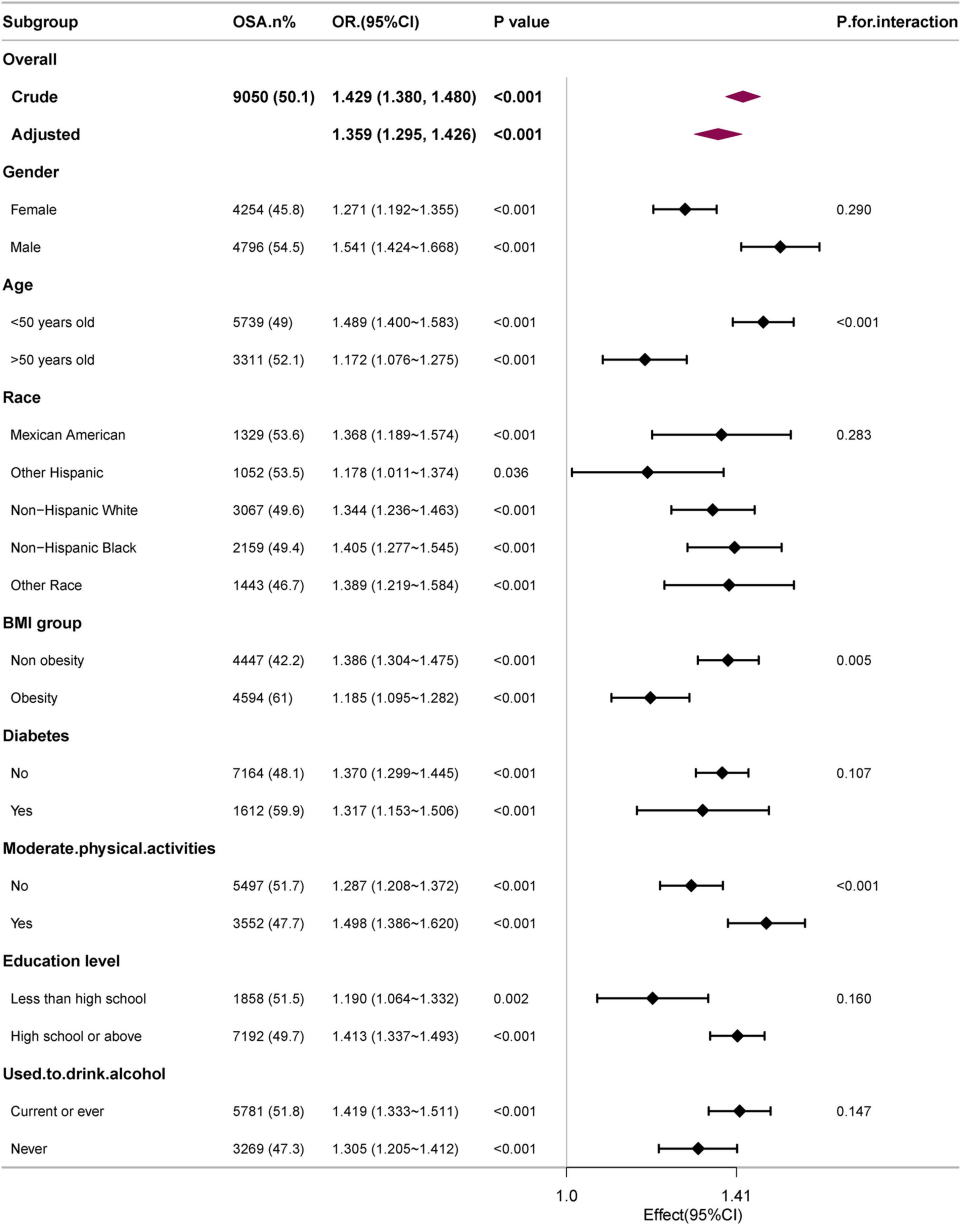
Subgroup analyses of the association between WWI level and OSA. All presented covariates were adjusted (as Model 3) except the corresponding stratification variable.

The robustness of the correlation between WWI and OSA was evaluated through comprehensive subgroup analyses and interaction tests, to identify potential population variations (Figure 3). The findings consistently indicated a significant correlation between WWI and OSA across most subgroups. Notably, there was a significant interaction effect between WWI and BMI, age, moderate physical activities (all interaction *p* < 0.05). The correlation between WWI and OSA was more pronounced in subjects with normal weight, younger and moderate physical activities.

### Non-linearity and threshold effect analysis between WWI and OSA

**Figure 4:**
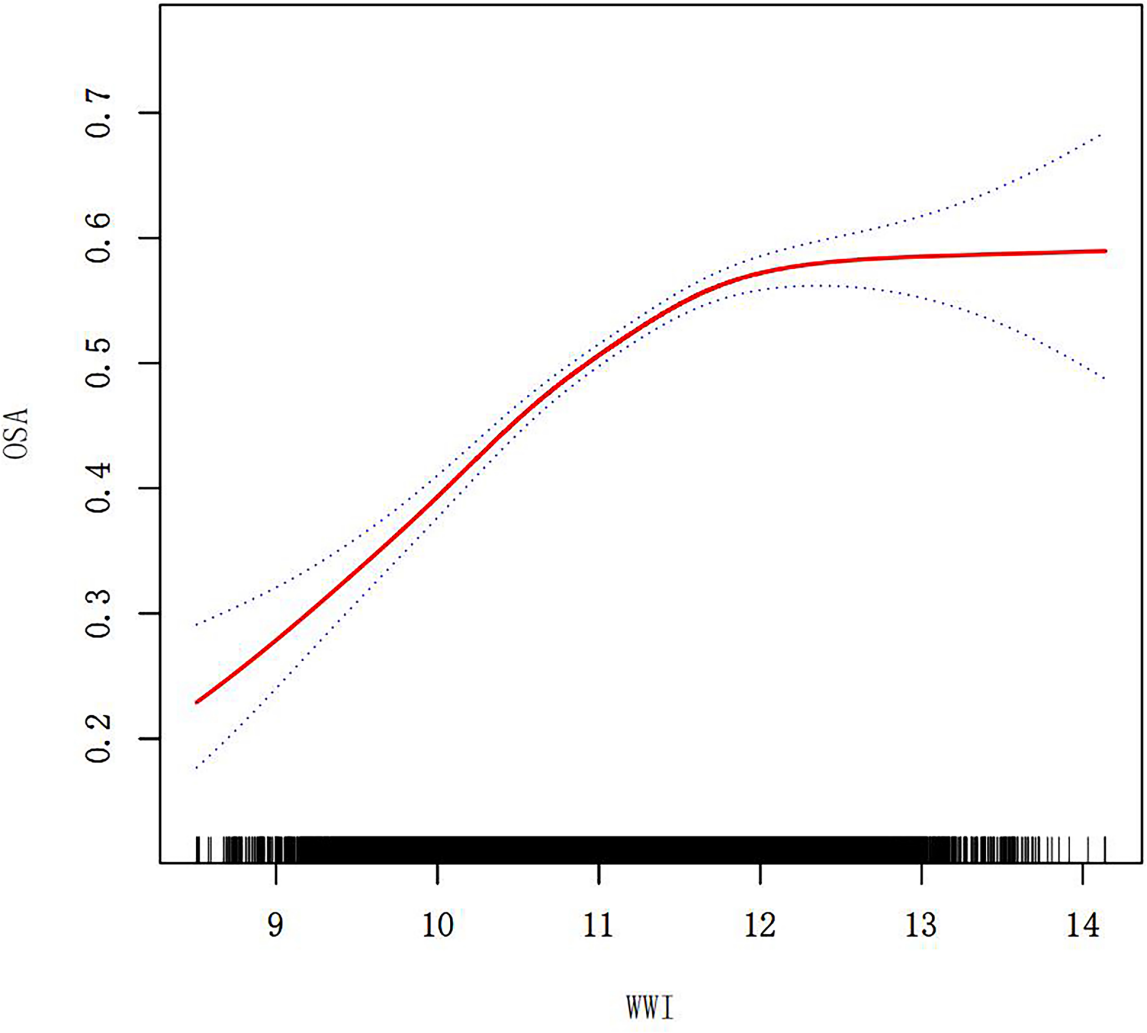
The smooth curve fit for the association between OSA and WWI.

**Figure 5:**
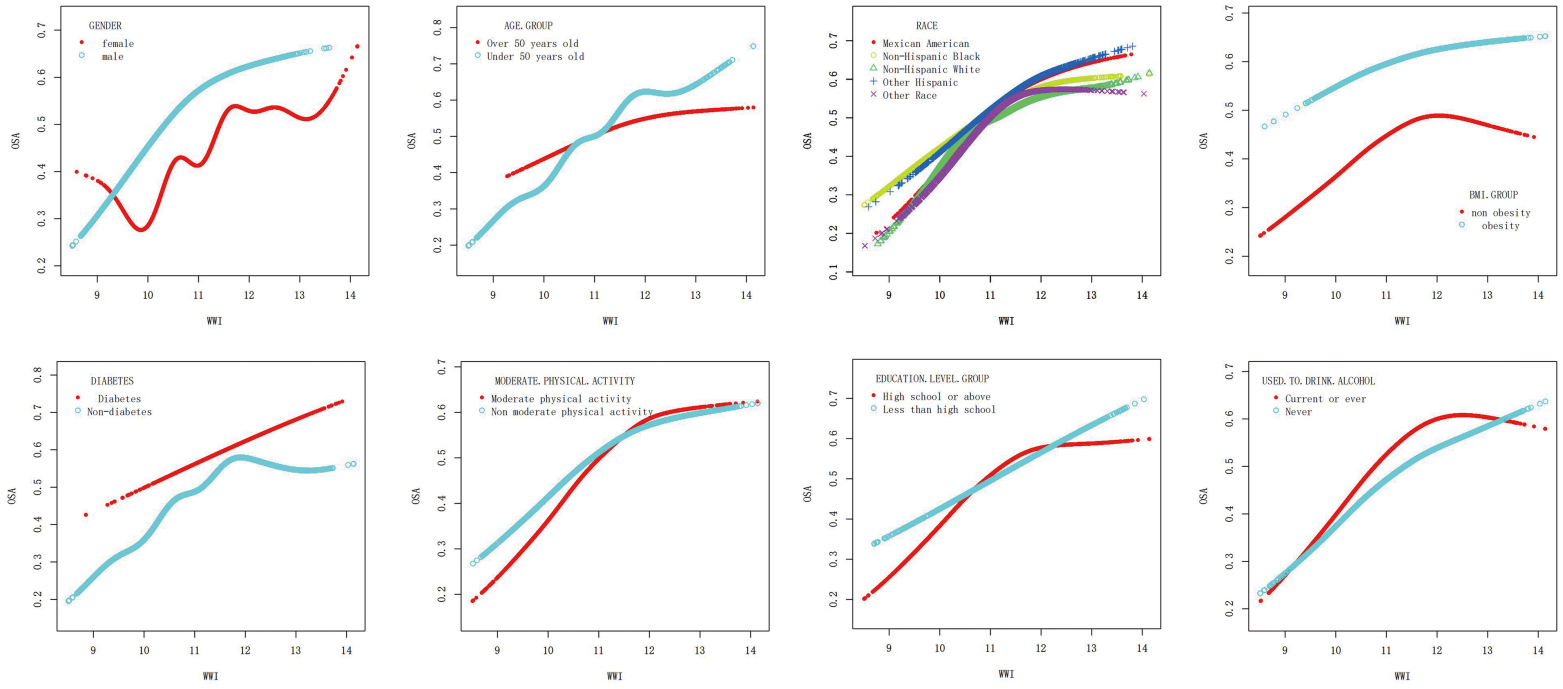
Association between WWI and OSA, stratified by gender, age, race, BMI, diabetes, moderate physical activity, education level and drinking. All presented covariates were adjusted (as Model 3) except the corresponding stratification variable.

To further explore the correlation between WWI and OSA, a smooth curve fitting analysis with Model 3 was conducted. The results depicted in Figures 4, 5 revealed a non-linear correlation between WWI and OSA. Through a subsequent threshold effect analysis, as detailed in Table 3, an inflection points for WWI at 11.678 (log-likelihood ratio < 0.001) was identified, indicating that when WWI level was below this threshold, there was a significant correlation with the risk of OSA. However, once WWI level surpassed 11.678, the correlation between WWI and the risk of OSA was diminished.

**Table 3:** Threshold effect analysis of WWI on OSA using the two-piecewise linear regression model.

WWI	Adjusted OR (95% CI), *p* value
Fitting by the standard linear model	1.359 (1.295, 1.426), <0.001
Fitting by the two-piecewise linear model	
Inflection point	11.678
WWI < 1.359	1.553(1.456, 1.656), <0.001
WWI > 1.359	0.993 (0.886, 1.114), 0.9088
Log likelihood ratio	<0.001

### ROC curves of each obesity indices

**Figure 6:**
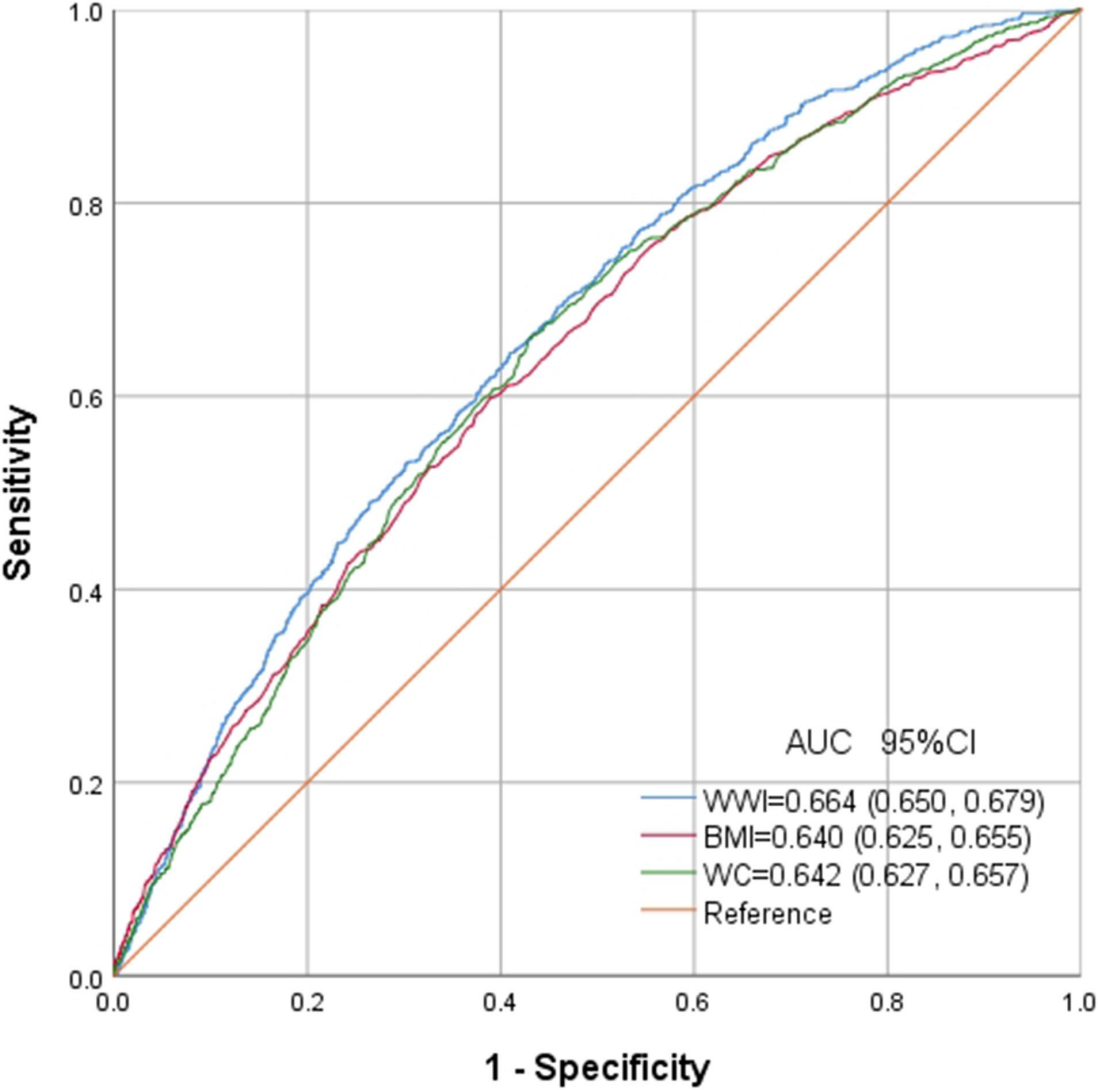
Receiver operating characteristic curves of WWI, BMI and WC to identify OSA.

ROC in Figure 6 presents the diagnostic performance of WWI, BMI and WC in identifying OSA. AUC for WWI in the ROC analysis was notably higher than that of BMI and WC at 0.664 (95% CI: 0.650, 0.679), with a sensitivity of 64.5%, a specificity of 59.1% and a cutoff of 11.3.

## Discussion

In this current cross-sectional study encompassing 18,080 representative adults, a notable positive correlation between WWI and OSA was identified. This correlation was particularly pronounced among individuals with normal weight, younger age, and moderate levels of physical activities. Notably, there was a non-linear correlation between WWI and OSA, with a saturation value of 11.678 identified across all participants. Furthermore, WWI exhibited a superior diagnostic accuracy for OSA compared to BMI and WC. These findings underscore the potential clinical importance maintaining optimal WWI in reducing the risk of OSA.

Epidemiological evidence suggests a potential link between traditional anthropometric measures and OSA, although the obesity paradox complicates this correlation. This paradox arises from the complex interplay among various anthropometric measures, obscuring the identification of biologically relevant disease risks (24). Consequently, it is crucial to differentiate accurately between lean body weight and fat mass. Furthermore, in contrast to emerging obesity indices based on intricate empirical mathematical models, WWI offers the benefit of simplicity in calculation, facilitating regular assessments within the broader population. Prior research has also demonstrated the efficacy of WWI in distinguishing fat and muscle mass, showing a negative correlation with muscle mass and a positive correlation with fat mass (25, 26). A longitudinal study spanning approximately 40 months revealed that CT data from 1,946 participants showed an increase in abdominal fat and a decrease in muscle measurements, accompanied by a rise in WWI (27). Ding et al. found a non-linear positive correlation between WWI and the risk of all cause and cardiovascular mortality in the China Hypertension Survey, independent of BMI and WC (28). Similarly, previous studies have also identified the role of WWI in predictor of multiple diseases, such as cardiovascular disease, hypertension, diabetes mellitus, hyperuricemia and metabolic syndrome, superior to BMI and WC (29–33). So far, it is the first study to assess the correlation between WWI and OSA. AUC of WWI was significantly higher than that of BMI and WC, suggesting the potential clinical utility for WWI in future clinical applications.

Subgroup analysis indicated that the correlation between WWI and OSA was notably stronger among participants under 50 (*p* < 0.001 for interaction). Consistent with the findings, Cai’s study demonstrated no significant correlation between WWI and overall mortality in individuals aged 75 years old and older (34). Additionally, Li et al. observed a link between WWI and hypertension in individuals under 60 years old in the Rural Chinese Cohort Study, which was absent in older adults (age ≥ 60 years old) (35). These outcomes may be attributed to differences in body fat distribution between younger and elderly people (36). Furthermore, individuals with normal weight exhibited a higher OR for OSA compared to those who were obese, suggesting that the population with normal weight may be more susceptible to the effects of WWI.

Furthermore, an intriguing discovery of a previously unreported non-linear correlation between WWI and OSA has been made. It is likely that there is a saturating effect of OSA when WWI reached 11.678 due to the fact that the prevalence OSA is already at a higher level in the high WWI population, and therefore, its changes are flatter. The findings have the potential to provide new insights into the treatment and prevention for OSA.

Several potential mechanisms may explain the positive correlation between WWI and OSA. WWI has been shown to accurately predict abdominal obesity regardless of overall body weight (25), leading to increased intra-abdominal pressure, reduced lung volume, and a higher risk of upper airway collapse (37). Additionally, the increase in WWI may reflect the accumulation of visceral adipose tissue, leading to the release of large quantities of pro-inflammatory factors, including leptin and aldosterone, resulting in oxidative stress and systemic inflammation. This phenomenon affects muscle function in the upper airway and facilitates the expansion of adipose tissue around the upper airway, thereby elevating the susceptibility to OSA (9, 38). Moreover, OSA itself can hinder weight loss efforts and contribute to weight gain. Disruption of hormonal regulation may occur due to the irregular sleep patterns and frequent awakenings correlated with OSA, leading to heightened appetite and cravings for calorie-dense foods (39–41). Furthermore, reduced energy levels and daytime fatigue can diminish the motivation for physical activities, exacerbating weight gain (42). The reciprocal correlation between OSA and obesity establishes a detrimental feedback loop in which each condition amplifies the other.

This study exhibits numerous methodological strengths that bolster its credibility and reliability. Specifically, the inclusion of the NHANES database guarantees a diverse and representative sample, closely adhering to database protocols. Additionally, a variety of regression models, including non-linear, linear, and logistic ones, were utilized in this study, to investigate the correlation between OSA and WWI. Additionally, the inclusion of subgroup analysis and interaction tests enhanced the validity and reliability of the results.

However, the study is limited by its cross-sectional design, which restricts the ability to observe dynamic changes in physical indicators, as well as its reliance on data solely from American adults, which may impede the generalizability of the results to other populations. Additionally, OSA was determined by the Berlin Questionnaire due to the lack of polysomnography during data collection, which is a commonly used validated tool in epidemiological and clinical research (43). Compared with many other lengthy and complicated screening questionnaires, the Berlin Questionnaire has been widely adopted and validated in various populations because of its ease to use, efficiency, and good sensitivity. Finally, despite comprehensive adjustments, the potential for unmeasured confounding factors exists.

## Conclusion

The current study demonstrated a positive and non-linear correlation between WWI and OSA, with a more pronounced effect of WWI on the risk of OSA among individuals with normal weight, those under 50 years old, and those engaging in moderate physical activities. These findings provided valuable new reference information for the risk assessment of OSA and added new evidence to its prevention.

## Data Availability

Publicly available datasets were analyzed in this study. This data can be found here: NHANES, http://www.cdc.gov/nhanes.
